# Supplementary material for: The Local and Electronic Structure Study of LuxGd1−xVO4 (0 ≤ x ≤ 1) Solid Solution Nanocrystals
Source: Nanomaterials (Basel). 2023 Jan 12;13(2):323. doi: 10.3390/nano13020323 (PMC9862454; doi:10.3390/nano13020323)
Supplement: Supplementary file 1 [file nanomaterials-13-00323-s001.zip › nanomaterials-2144039-supplementary.pdf]

Supporting information for

The Local and Electronic Structure Study of $Lu_xGd_{1-x}VO_4$ ($0 \le x \le 1$) Solid Solution Nanocrystals

Yang Chen [1], Ziqing Li [2], Nianjing Ji [3], Chenxi Wei [4*], Xiulan Duan [1*], Huaidong Jiang [1,4*]

[1] State Key Laboratory of Crystal Materials, Institute of Crystal Materials, Shandong University, Jinan 250100, China

[2] Institute of Optoelectronics, Fudan University, Shanghai 200433, China

[3] School of Materials Science & Engineering, Shandong Jianzhu University, Jinan 250101, China

[4] Center for Transformative Science, ShanghaiTech University, Shanghai 201210, China

[*]Correspondence:

weichx@shanghaitech.edu.cn (C.W.);

xlduan@sdu.edu.cn (X.D.);

jianghd@shanghaitech.edu.cn (H.J.).

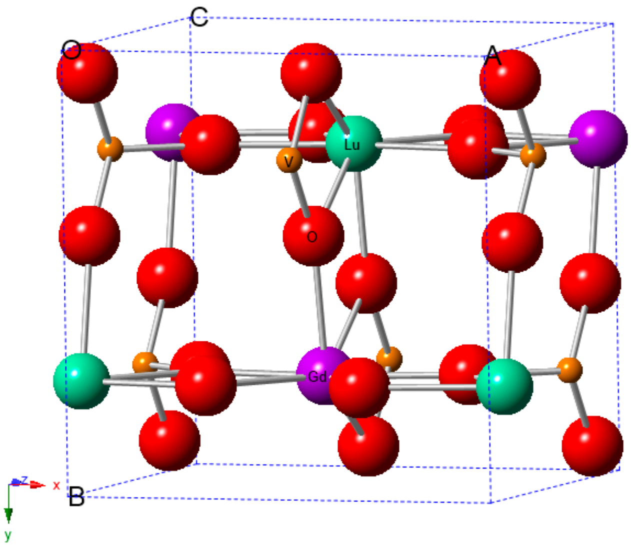

Figure S1. Unit cell model of $Lu_{0.5}Gd_{0.5}VO_4$.

Table S1. Rietveld refined cell parameters and R factors for $Lu_xGd_{1-x}VO_4$ solid solutions.

x_{calc}	*a* (Å)	*c* (Å)	*V* (Å^3)	Bragg R-factor (%)	Rp (%)	Rwp (%)
**0**	7.21287	6.35552	330.649	5.25	5.29	6.64
**0.1**	7.19318	6.34168	328.130	3.48	4.84	6.18
**0.3**	7.15467	6.31936	323.484	8.87	5.68	7.24
**0.5**	7.12131	6.29779	319.380	3.40	5.91	7.47
**0.7**	7.08135	6.27193	314.509	3.39	6.03	7.81
**0.9**	7.04636	6.25044	310.342	4.85	7.54	9.53
**1**	7.02852	6.23600	308.059	8.11	9.85	12.8

Table S2. Fitting results of the nearest and next nearest neighbor coordination shells of the FT-EXAFS spectra of $Lu_xGd_{1-x}VO_4$ at the Gd L_3-edge, the coordination number (*N*) was fixed.

x	Gd···O1.1			Gd···O1.2		Gd···O	Gd···V			S_0^2	R-factor (%)
	R(Å)	σ^2(Å^2)	ΔE_0(eV)	*R*(Å)	σ^2(Å^2)	*R*(Å)	*R*(Å)	ΔE_0(eV)	σ^2(Å^2)		
0	2.339±0.025	0.0054	3.6	2.457±0.029	0.0069	2.398±0.027	3.201±0.013	7.2	0.0035	1.0	0.83
0.1	2.328±0.017	0.0045	3.2	2.455±0.021	0.0057	2.392±0.019	3.197±0.012	7.0	0.0035	1.0	0.70
0.3	2.326±0.015	0.0044	3.4	2.454±0.021	0.0060	2.390±0.018	3.200±0.012	8.4	0.0036	1.0	0.83
0.5	2.301±0.014	0.0036	2.1	2.444±0.016	0.0040	2.372±0.015	3.187±0.012	6.3	0.0034	1.0	0.82
0.7	2.318±0.015	0.0034	3.8	2.458±0.022	0.0050	2.388±0.018	3.181±0.014	6.6	0.0033	1.0	1.3
0.9	2.280±0.020	0.0028	1.4	2.434±0.022	0.0020	2.357±0.021	3.173±0.023	4.9	0.0030	1.0	1.7

Table S3. Fitting results of the nearest and next nearest neighbor coordination shells of the FT-EXAFS spectra of $Lu_xGd_{1-x}VO_4$ at the Lu L_3-edges, the coordination number (*N*) was fixed.

x	Lu···O1.1			Lu···O1.2		Lu···O	Lu···V			S_0^2	R-factor (%)
	R(Å)	σ^2(Å^2)	ΔE_0(eV)	*R*(Å)	σ^2(Å^2)	*R*(Å)	*R*(Å)	ΔE_0(eV)	σ^2(Å^2)		
0.1	2.296±0.015	0.0032	8.1	2.417±0.039	0.0093	2.356±0.027	3.135±0.012	5.1	0.0022	0.91	2.1
0.3	2.266±0.017	0.0048	6.0	2.396±0.025	0.0079	2.331±0.021	3.152±0.012	7.8	0.0040	0.89	0.85
0.5	2.263±0.010	0.0049	5.8	2.397±0.018	0.0087	2.330±0.014	3.153±0.008	8.5	0.0039	0.93	0.77
0.7	2.251±0.008	0.0045	5.3	2.394±0.013	0.0076	2.322±0.011	3.149±0.008	8.7	0.0042	0.94	0.61
0.9	2.244±0.009	0.0048	5.1	2.386±0.016	0.0077	2.315±0.012	3.136±0.008	7.5	0.0040	0.92	0.77
1	2.242±0.006	0.0045	5.0	2.386±0.012	0.0076	2.314±0.009	3.134±0.006	7.7	0.0036	0.91	0.42

Table S4. Average distances from the dodecahedron central atoms to the first and second coordination shells and the dodecahedron distortion indexes for $Lu_xGd_{1-x}VO_4$ solid solutions.

x	Average distances		Dodecahedron distortion index	
	**RE⋯O (Å)**	**RE⋯V (Å)**	**GdO_8**	**LuO_8**
**0**	2.398±0.027	3.201±0.013	0.0246	
**0.1**	2.388±0.020	3.191±0.012	0.0266	0.0257
**0.3**	2.372±0.019	3.186±0.012	0.0268	0.0279
**0.5**	2.351±0.014	3.170±0.010	0.0301	0.0288
**0.7**	2.342±0.013	3.159±0.010	0.0293	0.0308
**0.9**	2.319±0.013	3.140±0.010	0.0327	0.0307
**1**	2.314±0.009	3.134±0.006		0.0311

The polyhedral distortion index was calculated by $D = \frac{1}{n}\sum_{i=1}^{n}\frac{|l_i - l_{av}|}{l_{av}}$ [1].

Table S5. Binding energies of characteristic peaks of all component elements and binding energy differences (ΔBE) between metal and oxygen characteristic peak of $Lu_xGd_{1-x}VO_4$ solid solutions.

x	0	0.2	0.4	0.5	0.6	0.8	1
O 1s (eV)	530.12	530.22	530.00	529.95	530.03	529.92	529.87
V^{5+} 2$p_{3/2}$ (eV)	517.32	517.40	517.18	517.14	517.22	517.11	517.07
ΔBE (O-V) (eV)	12.80	12.82	12.82	12.81	12.81	12.81	12.80
Lu 4$d_{5/2}$ (eV)	-	196.76	196.41	196.40	196.36	196.24	196.13
ΔBE (O-Lu) (eV)	-	333.46	333.59	333.55	333.67	333.68	333.74
ΔBE (Lu $4d_{3/2}$-$4d_{5/2}$) (eV)		9.89	9.92	9.84	9.93	9.93	9.93
Gd 4d (eV)	141.66	141.79	141.47	141.55	141.50	141.48	-
ΔBE (O-Gd) (eV)	388.46	388.43	388.53	388.40	388.53	388.44	-

1. Baur, W.H. The geometry of polyhedral distortions. Predictive relationships for the phosphate group. *Acta Crystallogr. Sect. B* **1974**, *30*, 1195-1215.
